# Entering Into the Story: Implications for Emergent Literacy

**DOI:** 10.3389/fpsyg.2021.665092

**Published:** 2021-11-25

**Authors:** Linda L. Sperry, Douglas E. Sperry

**Affiliations:** ^1^Dean’s Office, Indiana State University, Terre Haute, IN, United States; ^2^Department of Social and Behavioral Sciences, Saint Mary-of-the-Woods College, Saint Mary of the Woods, IN, United States

**Keywords:** oral narrative, emergent literacy, diverse families, language socialization, culturally sustaining pedagogy, early childhood, home-school transition

## Abstract

In this article we explore the ways in which three young children from a non-mainstream cultural group created stories with the assistance of their caregivers and siblings in the social contexts of their homes. We assert that these children’s oral narrations show us important dimensions of early experience with decontextualized content as practiced in their families that may offer suggestions for analysis of culturally sensitive experiences with literacy for all children. The dimensions we highlight are the *tangibility* of the elements around which the story is created, the *interlocutor support* children receive for beginning and continuing their stories, and the interaction between the storytelling process and the child’s *self-interest*. These three dimensions illustrate how children “enter” into stories and storytelling and broaden our understanding for fostering culturally sustaining pedagogy within schools.

## Introduction

When she was 5 years old, the daughter of the authors went on a sign-writing spree. Signs appeared on her bedroom walls and door saying, “Please do not come in my room unless I say,” and announcing that “Christmas is in 14 weeks” and “Halloween is in 6 weeks.” She pointed to them with pride, referred to them frequently, and used literacy to communicate her own affective concerns to others. She did not choose to author her own stories in the model of Babar, Dr. Seuss, Madeline, or any of her many other beloved picture books. Rather, she routinely chose to use literacy as a tool to reflect her socioemotional world (including upcoming holidays, and importantly, an 11-year-old brother who had recently posted a sign on his bedroom door forbidding entrance to all 5-year-old females). Enabled through her past and present experiences with literacy, *and* by her emerging knowledge of the social world, she constantly experimented with bringing literacy into her everyday life by framing her self-interests as written words and posting them on the walls.

Public education has as its fundamental purpose teaching all children to read and write. For years, teachers and publishers have promoted books and traditional book reading events as the primary means for provoking children’s interest in reading and writing and have encouraged parents to read to their children (e.g., Dickinson and Smith, 1994; Sénéchal et al., 1996; Whitehurst and Lonigan, 1998). However, Heath (1982), Philips (1983), Meier (2000), and Genishi and Dyson (2009) have pointed out the disconnections that exist for some groups of children, including, but not limited to, misunderstandings of the content of traditional emergent literacy practices and of the interactions within which classroom sharing time or book reading experiences occur (Michaels, 1981; Hicks, 1991).

As the opening example demonstrates, children from every cultural or socioeconomic background have their own self-interests that intersect with the societally promoted goal of having every school child learn to read and write. Unfortunately, these self-interests may not always parallel the instruction provided them in traditional school models. To remedy this discrepancy, we propose that it may be more productive to explore and build on what is *already* happening in the homes of children who come from diverse backgrounds. By adopting this stance, children’s home experiences can be examined to inform us of the multiple pathways that children may follow toward the eventual goal of learning to read and write. This stance is more pragmatic than using a deficit paradigm approach (Connor and Craig, 2006), which assumes children are behind from the beginning.

We do not seek to dismiss the views of scholars such as Delpit (1988), who has argued that minoritized children should be taught the language of power in order to take full advantage of the social capital accrued by such instruction. Indeed, they should. Rather, we aim to suggest that *all* children should learn about other cultures and practices in *significant* classroom experiences that *engage* those practices (cf. Paris and Alim, 2014) in ways that perpetuate and foster cultural pluralism (Paris, 2012). When we observe how *all* children interact with language within the context of their family life, we may begin to take advantage of a larger array of “teachable moments” than would be available if we concentrated solely on one activity of reading books or the practices of one social group.

Nevertheless, we are sensitive to the vast array of cultural backgrounds represented by children within our schools. The diversity of experiences and practices brought to school by children necessitates the sort of “bottom-up” approach anticipated by the culturally sustaining pedagogy proposed by Paris and Alim (2014), an approach that recognizes and honors each child’s abilities in turn. This more inclusive view requires us to understand the journey to literacy not as a single route but rather as a variety of pathways toward a shared destination. To navigate these pathways, we suggest looking not at landmarks that might be present on some roads but not on others (for example, early coaching of decoding skills or picture book reading), but rather at the universal motivations that are at the heart of each learner’s desire to begin the journey. To identify these motivations, we begin with personal storytelling which occurs very broadly across cultures (Schieffelin, 1990) and may be a cultural universal (Miller, 1994). In addition, personal storytelling is also understood by researchers as a practice that has been shown to predict emergent literacy (Feagans and Haskins, 1986; Curenton, 2006; Gardner-Neblett and Iruka, 2015).

The goal of having all children learn to read and write is the destination on the map of primary schooling; emergent literacy methods are the means of getting to the destination. For example, it is understood that phonological awareness, print recognition, and use of decontextualized language are key points along the journey (Snow et al., 1998; Curenton and Justice, 2004; Poe et al., 2004; Rowe, 2019). Although there is no one starting point of the journey toward emergent literacy, it is generally assumed to begin within the child’s language acquisition processes that occur within the context of family interaction (Roberts et al., 2005). To that end, although there are many objective features of language prerequisite to literacy, each of these features is learned in a very subjective social world. Wang et al. (2021) described how studying language as a social practice can make the linguistic strengths of children in minoritized communities visible, a point echoing the work of Miller who has consistently demonstrated that the narrative skills low-income children bring to the classroom may outstrip those of their middle-income counterparts (Miller et al., 2005; Miller and Sperry, 2012).

Children spend most of their young lives in the presence of their everyday caregivers and are immersed in the conversation that occurs there. Some of this conversation is about displaced events, which can be classified as either narrative or expository, and for that reason it is plausible to begin understanding the acquisition of literacy by the examination of narrativelike displaced-event talk. Narrative (as opposed to expository) displaced-event talk is probably the more common phenomenon within family conversations (Labov and Waletzky, 1967; Polanyi, 1989; Bruner, 1990). Children as young as 2 years of age engage in narrativelike displaced-event talk (Miller and Sperry, 1988; Sperry and Sperry, 1996).

Furthermore, personal storytelling has been linked to emergent literacy outcomes (Feagans and Haskins, 1986; Snow and Dickinson, 1990; Curenton, 2006; Gardner-Neblett and Iruka, 2015), largely through its role as a significant venue for the use and understanding of decontextualized language which predicts academic achievement (Rowe, 2012). The practice of storytelling capitalizes on children’s ability to define their own experiences and stories, an entry point to their understanding of narrative development and their eventual emergent literacy (cf. Dyson, 1997; Genishi and Dyson, 2009). Adair (2014) discussed the importance of fostering childhood agency in the classroom to allow them the “time, space, and opportunities to experiment and discover” (p. 232). One potential opportunity for the synthesis of emergent literacy practices and childhood agency is offered by the study and use of various storytelling practices within the homes of diverse families.

In sum, we emphasize young children’s oral language about topics displaced from the here and now because of its universality and because of its natural affinity with decontextualized language. Furthermore, it permits us a culturally focused view on the home language practices children bring to the classroom. Therefore, displaced-event narrative provides the potential for a useful resource for the construction of culturally sustaining classroom practices.

As we search for ways within early literacy instruction to connect it to our understanding of children as unique individuals, who come from particular cultural backgrounds and who exist at certain developmental levels, we must develop an awareness of children’s everyday experiences and the ways in which they author “texts” everyday (Dyson, 1997, 2003). This awareness is of keen importance for it places the focus of our investigation on the child’s lived experiences and not on our conceptualizations of storytelling practices. This focus is needed due to the likelihood of a home–school mismatch (Genishi and Dyson, 2009; Miller and Sperry, 2012) between literacy practices in the home and expectations in the classroom for many poor children and children of color. For example, in the Abecedarian project in North Carolina, African American preschoolers told longer and more interesting stories than European Americanpreschoolers within their homes and communities, but they did not experience any benefit from that prowess in terms of emergent literacy outcomes measured within schooling contexts (Feagans and Haskins, 1986). This and other paradoxical results (e.g., Corsaro et al., 2002; Dyson and Smitherman, 2009) call us to question the extent to which the various storytelling practices within the homes of non-mainstream children coalesce with the prevailing norms of the classrooms that children attend. We assert that literacy acquisition will best occur around content that is intrinsically interesting to the child, and, that to the extent that early narrative is related to literacy development, the most frequent topics of narrative will provide the best lens through which to view this development.

We have been immersed in the language use and practices of one cultural group of African American families from the rural South in the United States since our early work describing the narrative practices within their homes (Sperry and Sperry, 1995, 1996, 2000). However, rather than focus on the uniqueness of these practices (that may or may not reflect current practices), we use the data to help illuminate storytelling that marks the child’s agency and self-interest in the stories, and that may therefore provide a look into common features that comprise the narrations of all children as they enter the classroom, regardless of their cultural background. To accomplish this goal, we identified three narratives in which the caregivers followed the lead of the children. Accordingly, the stories represented the children’s own interests, which was additionally confirmed by the large number of child-initiated morphemes and overall length of the displaced event-centered talk. Through our reiterative microanalysis of three fantasy narrations, three avenues of approach to the culturally constituted ways these children entered into their stories emerged: *tangibility* of reference, the *interlocutor support* the child receives while the story is told, and the evocation of *self-interest* as the teller invests in the narrative. While we only address the instantiation of these ways within one particular group of African American families living in the rural South of the United States, it is our hope that these analytical categories will provide a tool that will be useful for entering into the storytelling practices of children from other cultural backgrounds (cf. Ballenger, 1999).

### Tangibility

Oral and written narratives are similar in that both refer to displaced events which have some quality of tangibility. For example, several studies have shown the efficacy of using specific experiences with picture books or informational books to help children create more complex stories (Pappas, 1993; Benson, 1997; Geist and Aldridge, 1999; Torr, 1999; Bavin, 2000). In a similar manner, the shared conversational practices within the cultural universal of personal storytelling (Miller, 1994) often consist of frequent caregiver contributions to jog the memories of very young children or to assist them in constructing the story in a culturally appropriate manner.

Some narrations use tangible props on occasion; for example, pretend play oral narratives specifically occur in the context of objects and agents who are transformed into other identities. Children’s ability to construct imaginary events and to participate in pretend play appears to be enabled by the physical artifacts present in their immediate environment—a broomstick “becomes” a horse; a box “becomes” a car (Vygotsky, 1976). In this way, the difficulty of maintaining completely imagined representations seems to be made easier by the presence of tangible objects in the immediate context. In that manner, tangibility might also be provided by other physical elements in the child’s environment such as a video or drawing, a storybook, a song or spoken poem, a familiar “story” often retold within the family, in combination with the gestures and stances of persons within the situated space (Goodwin, 2000). Any or all of these sources may be important in anchoring a narrative.

The topic of the narrative does not have to be anchored in the real world to achieve a sense of tangibility, however. Sperry and Sperry (2000) discussed how fantasy elements within the stories told by their African American participants may provide a type of zone of proximal development (Vygotsky, 1978) where caregivers granted young children wide latitude in talking about events that were understood by the caregivers to be impossible in the real world. In this manner, necessary elements of storytelling could be encouraged without requiring that each detail of the story be spatiotemporally accurate (a requirement that was strictly enforced within this community for stories of personal experience). In our following examination of the fantasy stories of three children from this community—Sebrina, Kendrick, and Stillman—we pay close attention to both the tangibility of the events as well as any physical elements involved in the social situation that help or hinder the children in their attempts to string together events to form a cohesive narrative.

### Interlocutor Support

Both oral narrative and literacy events occur in real time within social settings. Storytelling is linked to emergent literacy via social interactions (Anderson et al., 1997). Beals and Snow (2002) looked at low-income families from the Boston area. They compared narratives told during family dinner time with narratives elicited by home visitors. The children were much more successful when narratives emerged organically from dinner time conversation as opposed to elicited conversations. Berman (1995) also found that Hebrew children ranging between ages 3 and 8 years search for collaborative conversations. Two-year-old children do not always tell stories to other kids, but they tell stories to adults because adults provide the prompts to start and continue the stories (Kuentay and Ervin-Tripp, 1997).

Family members provide support along with the models for how to tell a story (Mardell, 1996). Children learn from their mothers how to talk and what to talk about during joint conversational experiences (Miller and Sperry, 1987; Hudson, 1990). The way mothers interact with children corresponds to the children’s current level of narrative skills (Wang, 2000; Minami, 2001; Cleveland and Reese, 2005). In a study of 10 children in Senegal, it was found that children’s access to dialogue routines that precede storytelling relies on assistance from adults and older siblings (Rabain-Jamin, 2001). Researchers agree that the 2-year-old child is unlikely to be able to fill in enough information for the story to exist as a narrated event in the absence of a knowledgeable interlocutor who participates with the child in the narration (Miller and Sperry, 1988; Nelson(ed.), 1989).

In sum, narrative making is a culturally constituted practice, imbued with the values, beliefs, and customs of the co-participants (Miller and Goodnow, 1995). Narrative practices are virtually inaccessible to incidental analysis due to their deep grounding in early, constant interaction between caregivers and children as they accomplish the “concrete routines of social life” (Schieffelin, 1990, p. 19, cf. Bourdieu, 1977). As such, any culturally sustaining pedagogy will demand significant effort in mining the richness of narrative practices each child brings to the classroom. With this in mind, we consider in this analysis how children, as individuals, manage their participation in producing their story, and how they manage their participation in the social event surrounding the story itself.

### Self-Interest

Third, both oral narrative and literacy events tend to flourish in the presence of self-interest (Paley, 1990). Given that the children whose stories are analyzed in this study are very young (approximately 2½ years old), their ability to construct a story is mediated by the participation of siblings and adults. In each case, the child constructed a fantasy in the company of a female caregiver (mother or aunt), a same-sex sibling, and the female researcher. However, in the construction, there is variability in the control they exert in maintaining their version of events in the story. McCabe (1998) recorded accounts of her child’s spontaneous productions. She concluded that although children needed others to create longer, more complex stories, her encouragement to continue or start a story only worked when it was what the child wanted. McCabe, like Paley (1990), suggested that stories are the manner in which children control and cope successfully with the reality of their lives.

Children need to control their creations because they must operate within the conventions that they have mastered (Crago, 1993); spontaneous creations require that children believe in their own solutions. Dyson (1991) studied eight students between kindergarten and third grade and found developmental differences in how much children controlled their imaginary stories and written worlds. Talk is needed as a tool in literacy development to cultivate imaginations. Often, we find ourselves trying to pry into children’s stories and fantasies by saying, “Tell me more,” or “What happened next?” However, children often start, stop, and extend the stories only if it is their own idea to do so. Children’s invented stories are transformative and are a means of discovering the sense children make of their experiences (Fox, 1998).

In sum, we hypothesized that three elements—tangible reference, interlocutor support, and self-interest—are paramount in how children participate in and learn about oral narrative. Furthermore, to the extent that oral narrative predicts literacy outcomes (Feagans and Haskins, 1986; Snow and Dickinson, 1990; Rowe, 2012; Gardner-Neblett and Iruka, 2015), these elements may provide useful tools to examine and compare the narrative productions of both mainstream and non-mainstream children with the goal of fostering culturally sustaining pedagogical practices (Paris and Alim, 2014). We suggest that these three elements likely form the foundation of all children’s stories, and therefore may provide an analytical prism to examine how all children “enter their stories” (cf. Ballenger, 1999). In studying the literacy development of Haitian preschoolers, Ballenger noticed that the children she worked with were eager to connect aspects of the stories she read to them to their own lives. During times when she read to the children, often the story reading had to be abandoned due to the children’s excited elaborations on their own tangential connections to items or events in the stories—and then to each other’s experiences. She referred to this intense identification with elements in the stories as “entering the story.” Specifically, entering the story seems related to the motivation for children to do the hard work necessary to develop emergent literacy skills.

Young children might enter a story of others through inventing their own stories, pretending, or singing. At the same time, in older children, the ability to enter into the story likely becomes an intrinsic motivation for choosing reading as an activity (Ballenger, 1999). It is also likely that children learn about story structure as they imitate and elaborate different aspects of stories: protagonists and antagonists, features of the setting, certain kinds of problems, successful resolutions. When young children enter into stories of their own making, they are able to gain important practice in using the basic elements of a story that surely relates to the motivation children will later use to conquer the difficulty of decoding in order to read stories in school.

It is our intent to offer rich descriptions of three different fantasy narratives that three children told in conjunction with their family members that embody all three elements we describe: the tangibility of the new stories as referents which recall previously experienced personal narratives; the interlocutor support family members provide children as they co-construct stories; and most importantly, the self-interest demonstrated by these children as they connect with the characters and events of the stories.

## Materials and Methods

### Research Setting

The community where these stories were collected was a geographically isolated, rurally dispersed community in the Black Belt region of the Deep South. The county within which the rural community was located was among the five poorest counties in one of the five poorest states in the United States. Historically, the data were collected between 1988 and 1990 (Sperry, 1991). The stories from this corpus have been analyzed numerous times (Sperry and Sperry, 1996, 2000; Miller et al., 2000, 2005; Miller and Sperry, 2012). While acknowledging that cultures are continually evolving, we suggest that, given our desire to consider analytical frameworks broad enough to offer insight into how all children enter into their stories, reiterative analysis of these narrations provides a useful resource for new insights for fostering culturally sustaining pedagogy (cf. Erickson, 2018). In this suggestion, we maintain a tradition of other longitudinal corpora studies within child language and anthropological research with respect to the validity of archived data for current analysis (e.g., Vidich and Lyman, 1994; Hart and Risley, 1995; MacWhinney, 2000; Zeitlyn, 2000; Erickson, 2018; Sperry et al., 2019). This tradition acknowledges the time- and labor-intensive nature of ethnographic data collection, transcription, and the rigorous description of community practices it affords.

### Participants

The participants were 14 African American families with 2-year-old children. All the families lived in the rural community. The socioeconomic status of the families varied, but the range itself was truncated. Some of the families were living in housing projects situated within small villages; some lived in houses and trailers on small farms carved out of the pine woods. The families also varied in terms of their household structure. The housing project families were all single mothers with their young children. The trailer families tended to be single mothers with children plus one or more young-adult relatives. The families who lived in houses tended to be associated with grandparents who were owners of the houses and who maintained an extended-family household that included their daughters and grandchildren. Only two of the 14 families were nuclear family households. Three of the families were biologically related to one another, but most of the families were socially acquainted with each other through the community.

### Research Design and Procedures

The design of this study was descriptive in purpose, ethnographic in approach, and longitudinal in terms of data collection. The researcher established herself as being interested in how young children learn to talk. Visits to each family’s home were made every 2 months as close as possible to the child’s birthday anniversary. The camera was always directed at the target child, and the camera and tripod were moved to accompany the child unless the child entered private areas of the house. Approximately half of the data collection occurred outdoors. Data collection was maintained until the child turned 3½ years old.

For this analysis, we followed common procedures in qualitative methodology (Strauss and Corbin, 1990; Janesick, 1994). First, we decided on the research question: how might we approach oral storytelling within this community in a manner that is true to the unique cultural practices within the community while potentially remaining true to storytelling practices common to children from other communities? In other words, might we devise an analytical framework that allows us to understand the stories of diverse children in order to capitalize upon this understanding within a culturally sustaining pedagogy?

Verbatim transcripts of half-hour segments from 70 observations (approximately 6 per child) were coded for displaced-event episodes consisting of at least one event not in the here and now stated by the child and one other on-topic utterance by the child. Episodes were then categorized as exhibiting fictional or temporal displacement, resulting in nine different genres. The three largest genres within the corpus were fantasy narrations (22% of all narrations), past narrations (23% of all narrations), and pretend narrations (28% of all narrations). We discriminated fantasy from pretend play based on the improbability of the narrated events in fantasy ever taking place in actuality. In other words, fantasies were constructed solely from mental and verbal resources (c.f. Shatz, 1984). By contrast, pretend episodes were defined as involving direct transformations of household items, toys, or people.

We chose fantasy narrations for analysis for several reasons. Fantasy episodes were initiated more often by children, they tended to be longer in length, and they contained more unique child contributions than the other genres of narrative (Sperry and Sperry, 1996). In this community, they provided fertile ground for socialization of self (Sperry and Sperry, 1995) and gender (Sperry and Sperry, 1996). Finally, they may have provided an important zone of proximal development for the development of narrative skills in general (Sperry and Sperry, 2000).

The requirements for selecting the specific three cases revolved around axes of similarity (Stake, 2008), including similarity of genre (all three were fantasies), similarity of social setting (all three involved more than one family member), similarity in age of children (all three are between 2½ and 3 years of age), and similarity in elaborated narrative (all three involved multiple turns by the child). The fantasy narrations selected for analysis in this study were quite representative of other fantasy narrations in the corpus. For example, these narrations shared thematic and performative similarity with the remainder of the corpus, the majority of which were produced in a playful stance. Typically these narrations related events about deliciously scary fictional characters, some common across multiple American cultural groups (the bogey man), some adopted from current media (Freddy Krueger), some variations of common animals or familiar people, and some made up on the spur of the moment and given unique, phonetically compelling names (e.g., Nicoudini, Scordini). For this analysis, we immersed ourselves in the stories and examined them holistically with attention to the sociocultural contexts of narration. At each stage, design decisions regarding the research questions, literature review, and data analysis were adapted through constant comparison and triangulation procedures until the interpretations were credible in terms of the participants’ lived experiences.

In presenting the talk of the children, their caregivers, and the researcher, we have organized the transcript into stanzas, defined as lines representing a topic, image, perspective, or theme (Gee, 1999). These stanzas are notated numerically, and each line—an idea unit recognizable through the stress placed on a particular word or phrase—is notated alphabetically. Speakers are indicated by one or two letters enclosed in parentheses. The contribution of the child to the stanza is indicated in bold font. Although we standardized phonology, we retained the words participants used and the order of those words in the utterances to honor the African American Vernacular dialect spoken by the families.

## Results

### Sebrina and the Bogeyman

Sebrina (age 2 years, 8 months) was playing outside in front of the small house she called home with her 4-year-old sister Kay, her adult Aunt Sharon (who was visiting from her home nearby), and the researcher, Linda. It was Christmas time, and, as Sebrina fixed her doll’s hair and dressed the doll, conversation lazily drifted across many topics appropriate to the season.

Aunt Sharon began to pose questions to Sebrina concerning Santa Claus’s impending visit with his reindeer. Eventually, Aunt Sharon mentioned a large deer which had entered the family’s yard earlier that day. Aunt Sharon interrupted her account of the deer to coax Sebrina to sing, “Rudolph, the Red-Nosed Reindeer,” a song that was a favorite in the community daycare that Sebrina attended sporadically. Sebrina appeared reluctant, and Aunt Sharon warned:

1a (Sh) Oh, you ain’t gonna sing. Santa Claus looking at you. See, that’s why Santa Claus’s deer came up in the yard to see what you was doing today.
1b (S) **No he didn’t**. (hands behind her head, smiling)

After Sebrina’s response to this fantasy scenario, Aunt Sharon continued to describe the deer’s enormous size and their pet dog’s reaction to it. This incident prompted Aunt Sharon to regale everyone with another story of a baby fawn that she and her brother had raised. At one point in the story, Aunt Sharon described how the baby deer would playfully butt various family members with its head. At last, Aunt Sharon related how the deer would interact with other visitors and commented that the deer “wasn’t afraid of nobody.” Glancing up at her aunt, Sebrina said:

2a (S) **I scared that boogie.** (Smiling) **I scared that boogie.**
2b (Sh) You did? And what did he do?
2c (K teasing) Eat her up.
2d (S) (Nodding) **Eat her up.**
2e (Sh) You scared that boogie bear away. Tell Kay. Didn’t you run him down the road?
2f (S) **Yeah.**

Aunt Sharon continued trying to elicit a story, but Sebrina answered only yes or no through several sets of conversational turns. However, Sebrina’s sister Kay then asked:

3a (K) Huh, Aunt Sharon, what that was, was in that well?
3b (S) **Boo—**
3c (Sh) That was the bogeyman. Ask Sebrina what it was. I ain’t got to tell you. Ask Sebrina.
3d (K) What it was?
3e (S) (Looking at K) **It was in that tree.**
3f (K) It was in that tree?
3g (S) **He gonna get you.**
3h (Sh) Sebrina, tell her what the bogeyman do to you when he gets you. He takes bad little girls. What does he do? He takes them away.
3i (S nods)
3j (Sh) He won’t bring ’em back home either, will he? (Pretending to be the bogeyman) “Oo-oh.”

At this point, Sebrina seemed willing to participate in the emerging fantasy narrative about the bogeyman, as did her sister Kay who, feigning fear, said:

4a (K) What, what, okay, is that him? That him? That’s him.
4b (S) (Standing up) **That boogie was right here. Standing right there**.
4c (K) That him? I heard something say, “Oo-oh.” (Looking around nervously) What was that?
4d (S) (Looking at Kay) **Bogeyman.**
4e (Sh) You heard Sebrina. She’s telling you what it was. He was out there scratching on her window one night trying to get in and get Sebrina, wasn’t he, Sebrina?
4f (S) (looking around) **He wanted to get in Mama’ door.**

At this point in the interchange, Sebrina seemed to become confused, and after a few more turns between Kay and Aunt Sharon, the fantasy topic was dropped.

### Commentary

#### Tangibility

The story-telling event took place outside Sebrina’s home, one of three small houses that faced a dirt courtyard in which some toys were scattered. Although these toys were a potential source for the creation of a story, in the social situation described above, Sebrina developed novel events about the bogeyman without the use of any physical artifacts.

In the preceding minutes before this episode, there was mention of the “bogeyman that was around the house the other day.” Aunt Sharon led Sebrina through a version of this story. However, in the first telling Sebrina never originated any portion of the story, merely repeating utterances previously stated by Aunt Sharon or Kay. In the present story Sebrina chose to return to an old topic, taking on the role of narrator. Her abrupt change of conversation back to an earlier topic may have functioned both as a way for Sebrina to encapsulate the preceding conversation and as a catalyst for her to control the ensuing narrative. Regardless, it is clear from the outset that Sebrina was employing remembered elements within the new story. These elements recalled at times fictional and at times actual events; nevertheless, at the moment of Sebrina’s entry into the conversation as narrator, the elements had no tangible support apart from the conversational context. Instead, her return to the previously told story, and her aunt’s encouragement of this return, demonstrated one way in which her new contributions were scaffolded (Sperry and Sperry, 2000). Allowing retellings of recent stories may enable young children to marshal discourse elements as they practice narratives they could not have begun or completed on their own.

#### Interlocutor Support

After Sebrina initiated the exchange with Aunt Sharon by saying, “I scared that boogie” (2a), Aunt Sharon quickly intervened with questions that may have assisted Sebrina in developing her story. Perhaps because of the fragility of the story that Sebrina began to construct, Aunt Sharon at times seemed to take control of the story to keep it going. However, in doing so, Aunt Sharon accomplished three tasks that scaffolded Sebrina’s construction of the story. First, she modeled the kind of questions that older (3 to 5-year-old) children might ask in seeking to clarify important elements of stories that they hear. Second, the questions posed by Aunt Sharon in turn might have helped Sebrina bring complicating elements into her story—Aunt Sharon asked, “You did? And what did he do?” (2b). Third, by asking these questions, Aunt Sharon proposed directions of development for Sebrina’s initial story (“You scared that boogie bear away. Tell Kay. Didn’t you run him down the road?” 2e).

Indeed, Kay, who is 1 year older than Sebrina, also asked questions to define the situation surrounding the story. Through her questions, Kay provided Sebrina with an opportunity to take back control of her bogeyman story. With each of Kay’s comments, Sebrina added another detail to the actions of the bogeyman—“It was in that tree” (3e), “He gonna get you” (3g), “That boogie was right here. Standing right there” (4b). Even while Kay directed most of her questions and comments toward Aunt Sharon, Sebrina brought the bogeyman closer and still closer to Kay until Aunt Sharon intervened again. In examining this interaction, Sebrina seemed to gain a sense of agency through recognizing her ability to affect how Kay interacted with her socially. This social interaction was both subtext and context for the story that Sebrina created.

Nevertheless, the most important interpersonal transactions in this episode occurred across all three participants. Sebrina’s episode presented the listener with an extremely complex interlocking of scaffolding by Aunt Sharon and Kay. On the one hand, both Aunt Sharon and Kay scaffolded the output of Sebrina. However, Aunt Sharon concurrently scaffolded the efforts of Kay as she attempted on the one hand to assist Sebrina’s contributions, and on the other hand to participate in the ongoing narration herself. At times, these complex scaffolding attempts crowded Sebrina out of the narrative construction, and her contributions to the narration seemed to be derailed. For example, early in the narrative, Kay interrupted Aunt Sharon’s effort to add a complicating element to the narrative (“And what did he do?” 2b) by her teasing comment, “Eat her up” (2c). Although Sebrina confirmed Kay’s remark in line 2d, Aunt Sharon effectively refuted Kay’s comment by asserting that Sebrina should tell Kay that she had, “…run him down the road” (2e). Nevertheless, the outcome of this elaborate interchange was effectively to derail Sebrina’s participation in the narrative momentarily as she appeared to become overwhelmed by the efforts of her aunt and sister and opted not to take sides in their ongoing competition to move the narrative along.

By contrast, Kay’s participation at times bolstered Sebrina’s efforts. Later in the episode, Aunt Sharon attempted to add interest to the story by pretending to be the bogeyman, making scary sounds. Kay immediately joined in the ruse, allying briefly with Aunt Sharon, as she turned to Sebrina to query, “Okay, is that him?” (4a). Her support of the play was made complete when she further stated, “That him” (4c), while feigning fear. This emotional support seemed all Sebrina needed in this case to pick up the narrative thread as she answered, “That boogie was right here. Standing right there” (4b).

It was not only the conversation of 4-year-old Kay that seemed to thwart Sebrina’s storytelling occasionally. At times, Aunt Sharon’s attempts at co-construction of the narrative threatened to silence Sebrina when her comments became excessively scary. For example, in the conversational turns immediately preceding the above interaction, the narrative almost came to a standstill when Aunt Sharon dwelled excessively on the fact that the bogeyman takes bad little girls away and will not bring them home (3h, 3j). This moment in the narrative truly brought to the foreground the importance of Kay’s conspiratorial interaction with Sebrina.

#### Self-Interest

Sebrina suddenly became part of this story when Aunt Sharon intervened to alter the direction of the story—“He was out there scratching on her window one night trying to get in and get Sebrina, wasn’t he, Sebrina?” (4e). In placing the bogeyman near to Sebrina, Aunt Sharon introduced a problem situation, to which Sebrina countered, “He wanted to get in Mama’ door” (4f). By moving the bogeyman away from her window, Sebrina, in a sense, found a way to resolve the problem in this story. Even at 2 years of age, Sebrina was beginning to participate, with the help of others, in shaping stories. Although the number of Sebrina’s responses was small, Sebrina accomplished multiple tasks in the exchange—she responded successfully to her aunt, and she interacted in novel ways with her sister. In this manner, she demonstrated her agency in the narrative process as she successfully initiated and retold her story about the bogeyman.

### Kendrick and the Abominable Snowman

Kendrick (2 years, 8 months old) and his brother Rodrick (almost 4 years old) were watching a video of the classic Christmas cartoon, “Rudolph, the Red-Nosed Reindeer.” In this episode, the video provided a wellspring from which flowed many oral narratives based on both past and fantasy events. For example, in the moments preceding the narrative presented here, the children’s mother, after seeing a bird’s nest on the video, related a story about an egg the boys had found the previous summer after it had fallen from its nest.

At one point, Kendrick moved toward the television set just as the Abominable Snowman reappeared on screen.

5a (M) Uh-oh. You better get back to your seat, fella.
5b (R) (Laughing as Kendrick runs back to the bench where he is sitting) He scared.
5c (K) (Shaking his head in denial) **Uh-oh. I got-. Him not gonna bite me.**
5d (M) Huh?
5e (R) (Laughing) He scar-, he scared Ken.
5f (M) Yeah.
5g (K) **I gon’ pop that big head.**
5h (R) (Laughing).
5i (M) You gonna pop it? What you gonna pop it with?
5j (K) (Nodding in agreement) **On that big head.**

The Abominable Snowman disappeared from the screen. Watching intently for a while, Rodrick, and then Kendrick responded, **“Uh-oh.”**

6a (M) Oh, where did he go?
6b (K) (Pointing at the TV) **What that monster? Him come out the yard?**
6c (M) Hm-mm, from behind a mountain.

At this point in the cartoon, the Snowman lumbered forward. Kendrick and Rodrick, watching intently, winced and screamed.

7a (R) What he trying to do?
7b (M) What’s that in his mouth?
7c (K) **In that monst-? In that monster? In that monster? Oh, in that monster teeth?**
7d (M) Yeah, he got some teeth. You want him to bite you?
7e (K) (Shaking his head no) **In him mouth?”**
7f (M) Mm-hm.
7g (K) **And some food.**

Rodrick continued commenting on the story action taking place on the television.

8a (R) Uh-oh. Look. Watch him fall down in that water. Watch! (screaming).
8b (K) (Looking at his mother) **Watch that monster.**
8c (M) Hm?
8d (L) Where’d he go?
8e (K) **Uh-oh.** **He gonna get down and touch you.**
8f (M) (Laughs) That-.
8g (K) **He trying to get that man.**
8h (M) Mm-hm.
8i (R) He lying in snow.
8j (K) (Turning to look at R) **No. I gonna pop that in the head.**
8k (R) (Looking at K) Who?
8l (K) **That monster.**
8m (M) But what-. Kendrick, what you gonna pop it with?”
8n (K) **That bone.**
8o (M) Huh?
8p (K) **Mm, that toy.**
8q (M) You’re gonna hit him with your toys?”
8r (K) (Shakes head “no”)
8s (R) (Sitting up on arm of the sofa) Pop him!
8t (K) **Pop it. I gonna pop that monst-. I’m gonna pop that monster.** (Shaking head) **That monster not going away.**
8u (L) Hm-mm (agreeing with Kendrick who looks at L).
8v (R) That monster going down in that water and then he went back up.
8w (K) **What that monster?**

In 8u and 8v, the researcher and Rodrick constructed an explanation of the story line to help Kendrick understand what has happened.

9a **(K) I see it and pop it.**
9b (M) You gonna pop it?
9c **(K) I’m [g]on’.**

The boys and their mother continued talking while the story reached its denouement with the Abominable Snowman being pushed off the cliff.

10a (K) **I ain’t gonna cry ’bout that monster.**
10b (M) You aren’t?
10c (K) (Shaking head). **I gonna pop that monster real fast. I gonna run over that monster. I gonna get my toys.**

### Commentary

#### Tangibility

Like Sebrina’s aunt, Kendrick’s mother participated in helping him to construct the story. However, unlike Sebrina, Kendrick had a tangible element around which his story was built—a television program playing on the screen. A sudden reappearance on the program of a monster redirected Kendrick’s attention to the television, and his mother brought Kendrick into the story by coaching appropriate behavior (“Uh-oh. You better get back to your seat, fella” 5a). From that moment, Kendrick’s attention and his words revolved both around what he was seeing on the program and around his mother’s questions. For example, when his mother asked what is in the monster’s mouth, Kendrick clarified by asking, “Oh, in that monster teeth?… In him mouth?” (7c, 7e). Paradoxically, it seems that the strong tangibility of the story also constrained Kendrick’s ability to take control of it. It is not until the researcher asked Kendrick, “Where’d he go?” (8d) that Kendrick began to direct explicitly the action of the story (“He gonna get down and touch you… He trying to get that man” 8e, 8g). Until that point, Kendrick’s main response to the threatening Snowman was that he’s “gon’ pop that big head” (5g, cf. 8j).

#### Interlocutor Support

At first glance, the interlocutor support provided within this episode seemed intertwined with the tangibility of the television show playing in the background. Kendrick’s mother was preoccupied with making comments relevant to the television show that simultaneously directed his attention to elements of traditional story structure. For example, Mother commented on information pertinent to the setting with statements such as, “Oh, where did he go?” (6a); she commented on information pertinent to the conflict posed by this antagonist with statements such as, “What’s that in his mouth?” (7b). Although Mother was asking questions about a fictional story, her concentration on elements derived from synchronous temporal activity was wholly consistent with a preference demonstrated by most mothers within the larger sample of this community from which these episodes are drawn. Mothers in general addressed over three times as many elicitations for temporal as for fictional information across the entire sample (Sperry, 1991). This determination to “get the facts straight” represented an important cultural value overall to these mothers while indexing significant aspects of narrative structure.

Kendrick’s mother, however, deftly followed his conversational attempts to enter into the narrative. Shortly after the episode began, Kendrick remarked, “Him not gonna bite me” (5c), a belief certainly grounded in his willingness to return to the bench at his mother’s direction. Immediately after this remark, his brother teased, “He scar’, he scared Ken” (5e, cf. 5b). Possibly in response to this tease, Kendrick commented, “I gon’ pop that big head” (5g). Mother selectively chose this act of agency on the part of Kendrick to assist him in the development of the resolution to the conflict presented by the monster. She immediately asked, “What you gonna pop it with?” (5i). After not receiving a satisfactory answer (and a temporary diversion by action on the television), Mother returned to this question toward the end of the episode. At this point, Kendrick suggested possibilities for the tool to carry out his action—first a bone (8n), and then a toy (8p) which appeared on the television.

In contrast to Aunt Sharon, Kendrick’s mother exerted more direct control over the conversational turns within this narrativelike episode. She subtly discouraged Rodrick’s interference in the conversation at times in an effort to focus attention on Kendrick (who she knew was the target participant in the study). Possibly for that reason, Kendrick’s contributions to the narrative seemed less compromised by the social interaction within the current context. Nevertheless, Rodrick’s occasional interjections often scaffolded the narration in unpredictable and productive ways.

#### Self-Interest

Even while Kendrick was emotionally invested in keeping track of the Abominable Snowman as a part of the television program, he imagined his own one-on-one encounter with the monster. And, it was by popping the monster that Kendrick displayed agency. He proposed to pop the monster from the very beginning and maintained his resolve to do this throughout the episode. By comparison with Sebrina’s wavering response to the bogeyman, Kendrick’s display of agency appeared immediate and unswerving. Perhaps this adopted stance was due in some part to Kendrick’s assurance that his compliance with his mother’s direction to “get back to your seat, fella” (5a) put him on the right side of the law. His self-interest preserved, he felt confident in his sustained participation in the narrative.

His almost 4-year-old brother and his mama encouraged his fantasy by treating his motive as reasonable, if not always his means (“You’re gonna hit him with your toys?” 8q). Nevertheless, he resolved to pop the monster and held fast to the solution of popping the monster on the head throughout a 3-min-long series of conversational twists and turns. He first suggested using a bone for hitting the monster, but when that suggestion was misunderstood, he nominated his toys. His mother criticized the idea of using his toys, and from that point forward Kendrick strengthened his resolve for quick and decisive action in the face of any threat: “I see it and pop it” (9a). A few minutes later once the Snowman has gone over the cliff, he recapitulated his plan: “I gonna pop that monster real fast. I gonna run over that monster. I gonna get my toys” (10c).

Perhaps the most interesting aspect of Kendrick’s story was the way that he switched from observing and asking questions about the actions of the Snowman on the television program to imagining his own interaction with the monster. After his mother questioned his decision to pop the monster with his toys, Kendrick reiterated his statement about popping the monster; in doing so, he placed himself as a protagonist in a confrontation with the Snowman. However, he also continued to make comments about what was happening on the program, and asked questions of his mother (“That monster not going away” (8t); “What that monster?” (8w). In this way, he explicitly demonstrated the interplay between the narrated event and the event of narration (Bauman, 1986).

In conclusion, one is aware from the beginning of the episode that Kendrick displayed a conversational competence that was not present in Sebrina’s narrations. The question arises whether this competence emerged from greater cognitive and linguistic skills, a heightened sense of socioemotional security, or a combination of both factors. The level of security was no doubt affected cognitively by the presence of two specific representational aids to Kendrick’s participation. First, the television program was available for additional information pertinent to the story being told. Second, few information processing demands were placed on Kendrick’s ability to narrate (cf. Shatz, 1984). This situation contrasted sharply with a narrative constrained in part by past memories (as in Sebrina’s fantasy), and in part by fictional elements which were being co-constructed by adult interlocutors into whose mental worlds the child must enter (as in Aunt Sharon’s version of what bogeymen do).

### Stillman and the Wolf

Stillman (3 years, 2 months) and his mother were drawing pictures on the coffee table for Granny and Auntie’s walls with paper and crayons provided by the researcher. Stillman’s 10-month-old baby brother, Avis, had just awakened from a nap and was sleepily sitting on the sofa. While Stillman debated about which crayon to use and what to draw, his mother, who was drawing a dog, announced:

12a (M) Ooh. This gonna be a big old wolf. Yeah, a wolf baby.
12b (S) (Interrupts his search for a crayon to stand up and look at Mom’s picture)
12c (M) This one of these prehistoric wolves.
12d (S) (pointing to her paper) **There go a wolf under there.**
12e (M) Yeah, I’ma try to make your brother a wolf.
12f (L) (to Avis)You’re getting a wolf, Avis?
12g (S) (in a worried tone) **Uh-uh.**
12h (L) (to Avis, and going along with Mom’s intent) I know you’re going to be scared. I would be.
12i (M) (handing the drawing to Stillman) Here you go. Show Linda your wolf.
12j (L) (speaking to Stillman) Lemme see it. Oo-oh, I’m scared of that wolf. Are you scared of him?
12k (S) **Huh?**
12l (L) Look at those big teeth he has. See those big teeth that wolf has?
12m (S) (studying the drawing) **Wolf [is a] dog.**

At this point, Linda suggested drawing bars around the wolf to put it in a cage, presumably to contain its ferocity. Mom drew bars for a cage as she discussed locking it up, which prompted Stillman to ask:

13a (S) **Why?**
13b (M) Why we put the wolf in a cage? So he won’t get out and get your little brother and you. When he tears the cage up, I’ma jump up and start running.
13c (S) **Huh? Ma, why? What put him in a cage for?**
13d (M) So he won’t get you.
13e (S) (posturing somewhat) **Well, weh-, I ain’t scared. That ain’t no wolf. I ain’t scared to see no wolf.**

Avis tears the drawing a bit. Stillman jumps up to stand over Avis in a somewhat threatening pose:

14a (S) (to Avis) **Wha’ that!** (then Stillman sits down again)
14b (M) (holding up her drawing) That’s a wolf in a cage.

Some discussion ensued about what Stillman wants to name the wolf.

15a (M) That’s a wolf in a cage.
15b (S) **What wolf in a cage for?**
15c (M) What your wolf gonna be named?
15d (S) (Pointing to drawing) **Name, “Dog.”**
15e (M) You’re gonna name the wolf, “Dog?” (laughs) A wolf is a type of dog, isn’t it?
15j (S) **I’m gon’ go grab him over by Grandad, gra-grab him by the, by the tail comin back.**
15k (M) You gonna scare that man with the wolf?
15l (S) **Eh. I gonna cut that cage off.**
15m (M) You’re gonna cut him out of the cage?
15n (S) **Eh. Then I bite him, gon’ bite, him.**

Avis begins crinkling the paper. Mother chastises Avis gently. The crumpled paper falls to the floor out of the baby’s reach. Stillman simultaneously contains his disappointment and tries to shame his brother**:**

16a (S) (to Avis) **Look! Mama made one wolf, look!** (looks down at the table, just glancing at Avis) **But Avis want it, all [of] it, and not quite big wolf.**

Stillman then lobbied to have his mother draw a new wolf for Avis, offering to get her a new piece of paper for the task.

17a (S) (to M) **I’ma get a piece.**

She ignored his request, and eventually the conversation turned to another topic.

### Commentary

#### Tangibility

Perhaps the most salient aspect of this fantasy transaction is the wolf drawing at its center with its large sharp teeth, yellow eyes, long tail, and black cage bars. The drawing emerged from his mama’s hand, and the paper containing the drawing was paper directly meant for Stillman’s use. Mama drew more interest to their mutual coloring project by suggesting that the dog she began drawing was actually a big wolf. She immediately mitigated the fear she was inducing by saying it was a baby wolf; still, she then intensified it by using the low-frequency word “prehistoric.” At first, the drawing was designated as a present for Avis, as Stillman’s mother commented, “I’ma try to make your brother a wolf” (12e). This prospect worried Stillman who said, “Uh-uh” (12g). Meanwhile, the researcher, in the spirit of participant observation, aligned herself with mama’s verbal play when she gently teased the baby, “I know you’re going to be scared” (12h). When the drawing was finished, Stillman and his brother both handled the paper, and this handling served to demonstrate how the paper served both as signified and signifier throughout the episode. While the talk of fear and the eventual drawing of bars around the animal prompted the participants to spin a free-flowing discussion of the wolf, Stillman was immediately brought back to the tangible fragility of the paper when his infant brother crumpled the drawing to Stillman’s dismay: “Wha’ that!” (14a). Finally, when Avis has crumpled the drawing, Stillman demonstrated his mastery over the purely symbolic nature of the paper by simply saying he will go get another piece of paper (17a).

#### Interlocutor Support

An equally critical feature of the episode was the dialectical nature of Stillman’s participation. From the outset, Stillman did not want to accept his mother’s interpretation of the events. His unwillingness had two components, emotional and social. Stillman reacted to his mother’s initial change of the emotionally pleasant dog to a more challenging drawing of a wolf by interrupting his drawing to join her (12b). He appeared at ease with his mother’s change of the situation until the researcher referenced her fear of the wolf, asking Stillman if he was afraid of it, too (12j). Stillman refuted the threat posited by the researcher with his doubtful, “Huh?” (12k). Then he contradicted the researcher by stating that the wolf is actually a dog (12m).

To mollify the scene, the researcher suggested that Mom put bars around the wolf. Unfortunately, this change to the drawing appeared to induce an effect opposite to that intended. At this point, Stillman resignedly plopped on the sofa next to his mother, and plaintively asked, “Ma, why?” in two separate conversational turns (13a, 13c). At last, Stillman resolved his fear by denying he was afraid coupled with an assertion that the picture “…ain’t no wolf” (13e). His denial was complete when his mother subsequently asked what he will name the wolf and he re-stated, “Name, ‘Dog”’ (15d). His mother confirmed his statement and reduced, whether inadvertently or deliberately, the level of threat posed by the wolf. Once Stillman’s version of the topic was accepted, his original contributions to the narrative increased.

His mama’s final alteration—adding the cage bars—set the stage for the majority of Stillman’s talk in the episode. He questioned why his mama drew bars over the wolf in three separate queries (13a, 13c, 15b). His questions offered his mother the chance to structure a plausible fantasy event sequence: “Why we put the wolf in a cage? So he won’t get out and get your little brother and you. When he tears the cage up, I’ma jump up and start running” (13b).

Despite his mother’s proffered narrative sequence, the specific fantasy events that Stillman initiated were unique and novel. They were not modeled in the researcher’s conversation, in the drawing, or in his mother’s speech. However, each action he invented was tied to one of the visual features of the wolf drawing: the tail (15j), the cage (15l), and the teeth (15n). Stillman used the visual references to weave a story line with himself as the protagonist–hero. Like Sebrina and Kendrick, at 3 years of age, Stillman was already well on his way toward being an accomplished oral narrator.

#### Self-Interest

Stillman’s sense of agency extended beyond the events of the fantasy to the social circumstances surrounding the narration. From the beginning of the episode, he responded with concern to his mother’s stated intention, “Yeah, I’ma try to make your brother a wolf” (12e). At first Stillman verbally expressed his opposition to her intention to give Avis the wolf picture: “Uh-uh” (12g). Although too young to lay claim to the drawing, the baby reached for the paper and when he tore a corner of it, his brother reacted with immediate alarm: “Wha’ that!” (14a), he cried as he jumped up to stand over the baby. Although the immediate threat of losing the drawing had passed, when the baby reached again for the paper, Stillman first tried to distract him, “Look! Mama made one wolf, look!” (16a). Then he appeared to console himself about the inevitable loss: “But Avis want it, all [of] it, and not quite big wolf” (16a). Stillman made one last pitch to his mother to draw a new wolf. When she failed to respond, the entire wolf topic was finally dropped.

Like Sebrina, who enacted the bogeyman at one point, Stillman enjoyed the idea of the wolf his mama created. As Sebrina’s aunt and Kendrick’s mother both did, Stillman’s mother entered the family into the fantasy (13b). With his narrated fantasy events, Stillman explicitly entered himself into the story: “I’m gon’ go grab him” (15j), “I gonna cut that cage off” (15l), “then I bite him” (15n). Unlike Sebrina, however, who appeared to become frightened when her aunt suggests that the bogeyman “…was out there scratching on her window one night trying to get in and get Sebrina, wasn’t he, Sebrina?” (4e), Stillman employed a tried and true solution of denial. He pronounced bravely, “Well, weh-, I ain’t scared. That ain’t no wolf. I ain’t scared to see no wolf” (13e).

## Discussion

What can we learn from Kendrick, Sebrina, Stillman and all children who come from backgrounds where “traditional” book reading practices may not be a part of everyday life? In looking across the three situations we have highlighted here, several issues are apparent.

First, oral narrative blossomed around tangible entities in the young child’s environment. These entities may be objects themselves, a situation that may be more analogous to pretend play than to the sorts of narrations we described here. However, the stories given above suggest that the tangibility of objects in the environment was often in a state of flux depending upon how the objects were used by the narration participants, and as such, elements of the imagination served to promote qualitatively different types of scaffolding within the narration (Sperry and Sperry, 2000). Although no physical artifacts were present in Sebrina’s story, the well-rehearsed events and characters themselves took on a near-tangible nature. However, when the imagined began to merge with the narration of actual events, Sebrina appeared to become overwhelmed by the potential tangibility of the bogeyman. In the stories of both Kendrick and Stillman, tangible referents do exist for the antagonists in the narrations, but the nature of these referents embodied salient differences, which impacted their usage by the children. The television story in Kendrick’s environment did not change with the whims of the narrative participants. Nevertheless, the cultural practice of encouraging fantasy narrations allowed Kendrick to place himself in the story without disturbing the fixed nature of its narrative. In contrast, Stillman’s co-narrators were freely changing the tangible referent in his story: first, the dog became a wolf, and then the wolf was put behind the bars of a cage. Finally, the referent itself was destroyed by his baby brother when Avis crumpled the paper. Stillman’s emotions vis-à-vis the referent likewise flowed freely as the tangible piece of paper changed throughout the episode.

Second, the participation of others (adults or children) in assisting children in telling their stories was a significant factor present across the three examples. To varying degrees, each adult took the role of question–asker, and attempted to help the child build on the story that was co-created, and it was this participation (along with the participation of siblings) that shaped how each child chose to construct their story. In Sebrina’s case, Aunt Sharon’s comments about the Bogeyman trying to get in Sebrina’s window drew Sebrina in as a character in her own story. When Kendrick’s mother questioned him about what he will use to pop the monster, this helped Kendrick to produce several possibilities. And, without the action of Stillman’s mother drawing a wolf, Stillman would not have produced a story revolving around his ability to defeat the wolf.

As the participation of adults in the story shaped what Sebrina, Kendrick, and Stillman created, the presence and participation of their siblings sustained the storytelling event. In each case, the sibling’s interaction with the child promoted expansion and longevity of the narrative conversation. This was perhaps most clearly apparent in Sebrina’s story when Aunt Sharon enacted the bogeyman, drawing both girls into the conversation. This was also clear in Kendrick’s case; his brother Rodrick helped Kendrick to enter the story when Rodrick first acted afraid of the monster. By maintaining attention to the events of the cartoon and commenting on these events, Rodrick perhaps encouraged Kendrick to maintain his attention to the program as well. And the presence of Stillman’s baby brother surely made plausible Stillman’s preoccupation with securing both the drawing and his mother’s attention for himself.

The participation of others forces the child to deal with multiple perspectives, another characteristic of successful reading comprehension. Sebrina was alarmed when her Aunt Sharon suggested that the bogeyman scratched on her window one night. She had to act fast, in this case to counter that “he wanted to get in Mama’ door.” Kendrick was kept in the story by the involved participation of his mother and brother, and after multiple requests from both participants to say what object he would use to pop the monster, his brother helped him out by refocusing on the importance of just popping it. Stillman, more than the other two preschoolers, was forced to deal with others adjusting the story line counter to his wishes. He safely named the wolf, “Dog,” when others insisted it was a wolf. He had to defend the drawing from his baby brother’s attempts to crinkle the paper. He struggled to defend his position that the wolf was a harmless dog, and finally succumbed to others’ insistence that the wolf was a menace by grabbing its tail, cutting the cage off, biting it, and neutralizing it. This practice with adjusting to others’ perspectives is surely helpful for learning to identify the multiple motives of story characters (Pellegrini et al., 1995).

Finally, in each case, the self-interest of the child moved directly to the foreground of the narratives analyzed. It should not be surprising that the toddlers wanted to talk about themselves. What is compelling about the narratives analyzed here is the particular direction that the self-interest of the child took in each case. All three narratives developed around themes of threats that needed to be conquered, and all three children in their own ways took on the threat in their specific situations. Sebrina, once she finally acknowledged the threat of the bogeyman, sought refuge from the threat by becoming part of the storytelling fabric itself, avoiding direct participation in the events of the story. One might say that she placed her bets in a safe alliance as co-narrator, effectively moving herself away from the scene of the action. On the other hand, Kendrick placed himself squarely within the confines of the story’s action, establishing himself as the lone knight who would conquer the Abominable Snowman. Despite his mother’s and brother’s attempts to impugn his ability, he persisted with his plans to “pop” that monster. Lastly, Stillman represented an interesting counterpoint to the above two examples. His self-interest was threatened at the level of both the story event and the storytelling event; both the wolf and his brother Avis challenged Stillman’s position of security. In the end, Stillman handled both evildoers with aplomb. On the one hand, he eliminated the threat within the story posed by the wolf when he announced, “I ain’t scared to see no wolf.” On the other hand, he dismissed the threat to the storytelling posed by Avis’s damage to the paper drawing by non-chalantly asserting, “I’ma get a piece [of paper].”

In sum, all the episodes analyzed featured the toddler accepting a threatening situation as an obstacle around which to form goal-directed behavior. While in each case the resolution of the conflict took subtly different forms, it must be noted that each child had already mastered the basic structure of successful narration: the identification of a conflict, the establishment of goal-directed behavior to resolve the conflict, and the purposeful movement toward the realization of that goal.

In the end, we argue that the support provided by these three elements—tangibility, interlocutor support, and self-interest—provided both the cause and the means for these children to “enter” into the story. Furthermore, we assert that these three elements provide a generic point of reference with which narrative productions of young children from other cultural groups might be analyzed. Experiences in the classroom that provide for variable student control over each of these dimensions may successfully stimulate interest and effort. Whitehurst and Lonigan (1998) distinguished between outside–in and inside–out skills. This dichotomy proves useful in ascertaining the parameters of approaching literacy from a culturally sustaining perspective. The oral narrative dimensions we describe here—tangibility, interlocutor support, and self-interest—connect most directly to outside–in skills. With the supported proffered by these dimensions, all three children were able to muster the information necessary to develop an oral narrative storyline, an inside–out skill. In a similar manner, it is not hard to imagine that these same outside-in dimensions might ease the burden of learning the inside-out skills of letter, word, and sentence decoding.

### Connections Between Home and Classroom

The participation of adults, the significant social interactions with other children, and the relative spontaneity and creativity involved in becoming part of one’s own story make us think seriously about how the cases above may be important for practicing teachers to examine as they engage in a culturally sustaining pedagogy (Paris and Alim, 2014). These elements are already aspects of every classroom—can we bring them to bear even more explicitly on the emerging literacy of young children?

The answer, of course, is yes. First, tangibility can be varied along a continuum from video or film sequences and storybooks where characters, settings, and events are spelled out straightforwardly to posters and photographs to drawings created by children themselves. However, based on the cases presented in this essay, we propose that tangibility needs to be defined foremost from the perspective of the child. The typical mainstream school answer to whether or not a story can be changed is typically a resounding “No”; this attitude carries into the classroom as mainstream teachers interact in sometimes unfortunate ways with diverse children (Michaels, 1981; Genishi and Dyson, 2009; Corsaro and Rosier, 2019). The mainstream literate world seems to demand a “literal” interpretation. Literate adults view the printed page as unchangeable and the stories upon the printed page as unvarying. Furthermore, we aggressively socialize young children into this belief system. For example, we place high value on the accurate construction of oral narratives of personal experience, correcting small variations in sequence of events or insisting that all elements of the narration be truthful. In school book reading circles, we often encourage children to read stiffly in order to say each word accurately rather than encouraging children to read fluidly with errors. In storytime at bedtime, we support children’s preferences to read the same story over and over again, reinforcing each time that there is one correct version of a particular tale. None of these examples represents a situation without merit toward attaining the goal of personal literacy. However, one reason why fantasy reference is more often child-initiated and child-sustained than past reference in the talk of these African American toddlers is because their families place such a high truth value on past talk, which [taking great care that their children do not become “storyliars” (Sperry, 1991)] acts to suppress young children’s efforts (Sperry and Sperry, 1996). Obviously, care and concern for the method of progression from a verbal to a written world must be taken. The printed word is often an unforgiving taskmaster. Yet, the cases presented here highlight the potential natural connection between the decontextualized world of personal oral narration and literacy, a connection that may be used beneficially within the school context.

Second, interlocutor support can be deliberately varied from highly structured interactions to innovative interactions, probably with the assistance of peers (Dyson, 1997). For beginning narrators, the interaction should probably be social. However, despite the social nature of each of these narratives, each child performed several functions in their telling, freely assuming the role of co-narrator and sharing the shifting authorship and perspective as the story progressed. We were reminded of Goffman’s (1980) production formats. In his seminal description of footing, Goffman described the various positionalities a speaker may take when delivering a message (animator, author, and principal).

The children in this report were never simply the animator (sounding box) of their stories; neither were they ever solely the author as might be expected in a typical fictional account. In each of these three stories, the children inhabited various roles as they either retold or corresponded with their co-narrators. This complexity of performance stands in stark contrast to the monologic nature of the traditional elementary school sharing time where the espoused goal is for the child to serve as animator of a story fixed in time and reality. In addition, it cannot be assumed that the particular production format typified within these stories is shared cross-culturally. The interplay between diverse young narrators, socialized into different styles of production formats, and their mainstream teachers may be at the root of many discursive misunderstandings in the classroom. This possibility needs additional attention.

Finally, children’s self-interest as demonstrated by their affective pull toward the material in their stories must be allowed to flourish. In giving young children varied opportunities to experience creativity with their stories as well as the stories of diverse others, it is inevitable that individual children will have social interactions with others that will expand their repertoire of possible story settings, complications, and resolutions. Adair (2014) defined agency in the classroom as “the ability to influence what and how something is learned in order to expand capabilities.” In general, the fostering of agency in the classroom allows children to bring to school their own funds of knowledge (Gonzáles et al., 2005). Agency in language learning allows children the “time, space, and opportunities to experiment and discover” the various language resources around them (Adair, 2014, p. 232). When children are allowed to make use of these resources, they are repositioned as experts in what they already know (Comber, 2016), which in turn grants them license to take chances in learning something they do not. It is not as if these resources are not in abundance in the modern classroom. Dyson (2016) presented multiple vignettes of the ways children from diverse cultural backgrounds negotiate the making of their own stories in the classroom, at times meeting with success and at times not. We hope that providing strengths-based terminology for emergent literacy, such as the importance for young learners of self-interest, tangibility, and interlocutor support, might help teachers frame their approach accordingly (Michaels, 1981; Genishi and Dyson, 2009; Miller and Sperry, 2012; Corsaro and Rosier, 2019). It is critical to avoid classroom situations where diversity goes unacknowledged which, in the process, runs the risk of relegating literacy for all students from the crowning achievement of the American educational system to a problem to be fixed (Miller and Sahni, 2016).

## Conclusion

When our son was 5½ years old, we moved to a new home several miles from the university where we both worked. The fastest route to and from work often took father and son past two institutions: a federal penitentiary and a home for troubled adolescents. Both facilities were situated on relatively large campuses and were quite imposing, intriguing to our son, and they called forth many questions. While attempting to find age-appropriate answers to his son’s many questions, the father often found himself resorting to making very general statements about the “bad” reasons that might explain why the residents of these facilities were currently housed within their walls without going into too much detail about the structural violence that permeates our society and contributes to incarceration.

One day, the son responded particularly anxiously to his father’s admonition about avoiding some particular “bad” behavior. The father will long remember the tearful anxiety of his son, however, as he sorted out the fact that the son thought that if he were “bad” he would have to reside in one of these institutions. Finally, the father, at an inept loss for words (while nevertheless finally realizing his responsibility for this scene), told his young son that while what was under discussion was indeed “bad,” his action was not “bad-bad” like those of the residents of the neighboring institutions.

This scene of personal family lore is recounted here to make a point that we consider essential to the analysis within this paper. Despite our best attempts to analyze the syntactic or semantic elements of a narrative, or by extension the phonetic or whole-language approaches to literacy acquisition, they will all miss their mark unless we attend seriously to the unique ways in which an individual child enters into and alters the story itself. It is for this reason that we propose that the construct of entering into the narrative is essential for understanding how children learn to impart meaning through their own narratives as well as derive meaning from those of others, and it is absolutely essential to a culturally sustaining pedagogy.

Bourdieu has offered us the perfect explanation for why the mismatch between home and school practices can hinder the language and literacy development of non-mainstream children. When one habitus collides with another, as in the case of non-mainstream children encountering mainstream educational practices, each habitus seeks self-preservation of a sort, a condition Bourdieu (1977) likened to *hysteresis*, the tendency of any system to depend upon its history to manage the present moment. For this reason, the non-mainstream child and the mainstream educator must carefully negotiate their interactions to avoid the nature of the habitus to sustain and perpetuate itself.

Fortunately, both child and educator often bring to the situation a mutually aligned disposition, namely the complementary desires to be educated and to educate. Corsaro and Rosier (2019) and Phelps and Sperry (2021) described how the African American mothers in their studies actively primed their children to prepare for school in material and emotional ways according to their understanding of the schooling context, stressing both the practical and moral value of academic pursuits. Melzi et al. (2020) demonstrated the willingness and desirability Latine families possess to engage in supplemental educational practices and school participation, and that these dispositions significantly impacted their children’s emerging narrative skills.

Unfortunately, despite best intentions, all parties involved often find it difficult to negotiate the differences between mainstream and non-mainstream dispositions. For example, the priming events described by Corsaro and Rosier (2019) backfired despite the well-meaning intentions of Head Start teachers of color preparing their students for mainstream elementary school. The Head Start teachers focused on the interactional styles they saw as hampering their students based on their interpretations of the rigors of elementary school. Therefore, they insisted on single, correct answers to ambiguous problems that the children were often solving equally correctly with creative analysis of the problem itself. Furthermore, children’s answers were deemed wrong when they were not expressed in complete sentences, a foregrounded requirement that obscured the meaning-making potential of the children’s responses. In sum, their desire to prepare their students for the foreignness of the mainstream classroom created uncertainty and anxiety in their students that, in turn, silenced the children as they became increasingly unwilling to volunteer answers.

What strikes us is that these educators were well-acquainted with the manifest practices of mainstream schooling, but not with latent dispositions underlying these practices. Without the understanding of the “why” behind a practice, they focused on rigorous adherence to a classroom practice in the future. Similarly, teachers in the mainstream setting, unaware of the dispositions of children of color, are subject to hysteresis, taking for granted the dispositions of their own socialization. This process is also consistent with Bourdieu (1977) who suggested that the longest held dispositions are the most likely to be resistant to change.

We argue it is important that teachers learn to recognize when children begin experimenting with connections between the factual world and the fictional world through entering the stories of their own and others’ making. We further argue that this recognition may be especially critical to facilitating the literacy development of students who come from backgrounds outside of mainstream culture. Within recent years, researchers have made a greater effort to understand what goes on in home and then bring that knowledge to the practice of teachers (Gonzáles et al., 2005). However, work on early literacy that *begins* in the homes of young children remains largely unaccomplished. Exposure to and analysis of stories such as these told by Kendrick, Sebrina, and Stillman bring forth the value of ethnographies of literacy that might be developmental in scope and pedagogical in focus. This analysis is only a small starting point; much work needs to be done to understand how children from other diverse backgrounds may employ the elements of tangibility, interlocutor support, and self-interest within their stories. Furthermore, the difficult work involved in engaging such an understanding in everyday teaching practices and curricula remains to be done. Nevertheless, to the extent that we “hear” the stories of these and other young children, we will become more effective in teaching them to read the stories of others and, eventually, to write their own individual stories. To the extent that we create familiar contexts of practice for all children, we will become more effective in helping children bridge the transition from listening and telling to reading and writing. In other words, we must become familiar with the lives of all children who enter our classrooms, and that familiarity comes at a price.

In this moment, there is emerging consensus that we as educators are aware of this price and are willing to pay it. But what are we buying? Are we buying simple prescriptions for parents to talk more to their children? Are we buying admonitions to surround non-mainstream children with more decontextualized talk? Unfortunately, these “fixes” remain grounded in the notion that non-mainstream practices are the problem and not the solution. We think the answer is, on the one hand, readily available, and, on the other hand, much more difficult than these current vogues for literacy advancement. Rather than finding simple “fixes” for non-mainstream children and their families, we as mainstream folks must do the work of learning about other cultures and practices in the same way as these non-mainstream actors are forced to do on a daily basis. We must immerse ourselves in the lives of others—we must enter into their story.

## Data Availability Statement

The original contributions presented in the study are included in the article/supplementary material, further inquiries can be directed to the corresponding author/s.

## Ethics Statement

Ethical review and approval was not required for the study on human participants in accordance with the local legislation and institutional requirements. Written informed consent to participate in the original study was secured from the participants’ legal guardian/next of kin. All names are pseudonyms.

## Author Contributions

Both authors listed have made an equal, substantial, direct, and intellectual contribution to the work, and approved it for publication.

## Conflict of Interest

The authors declare that the research was conducted in the absence of any commercial or financial relationships that could be construed as a potential conflict of interest.

## Publisher’s Note

All claims expressed in this article are solely those of the authors and do not necessarily represent those of their affiliated organizations, or those of the publisher, the editors and the reviewers. Any product that may be evaluated in this article, or claim that may be made by its manufacturer, is not guaranteed or endorsed by the publisher.
